# Retinal Vascular Density as A Novel Biomarker of Acute Renal Injury after Acute Coronary Syndrome

**DOI:** 10.1038/s41598-019-44647-9

**Published:** 2019-05-30

**Authors:** Guillaume Alan, Charles Guenancia, Louis Arnould, Arthur Azemar, Stéphane Pitois, Maud Maza, Florence Bichat, Marianne Zeller, Pierre-Henri Gabrielle, Alain Marie Bron, Catherine Creuzot-Garcher, Yves Cottin

**Affiliations:** 1 Cardiology Department, University Hospital, Dijon, France; 2 PEC 2, Univ. Bourgogne Franche-Comté, Dijon, France; 3 Ophthalmology Department, University Hospital, Dijon, France; 4 INSERM, CIC1432, clinical epidemiology unit, Dijon, France; 5 Dijon University Hospital, Clinical investigation Center, Clinical epidemiology/clinical trials unit, Dijon, France; 6 Eye and Nutrition Research group, CSGA, UMR 1324 INRA, Dijon, France

**Keywords:** Interventional cardiology, Acute kidney injury, Predictive markers

## Abstract

Iodinated contrast agent (ICA)-induced acute kidney injury (AKI) following acute coronary syndrome (ACS) is a frequent complication, which may lead to chronic kidney disease and increased mortality. Optical coherence tomography angiography (OCT-A) of the retina is new tool delivering a rapid and noninvasive assessment of systemic microvascularization, which is potentially involved in the occurrence of ICA-induced AKI. Between October 2016 and March 2017, 452 ACS patients were admitted to our cardiac intensive care unit. OCT-A was performed within 48 h after the ICA injection. Patients with a history of retinal disease were excluded. The patients included were divided into two groups depending on whether or not AKI occurred after injection of ICA, according to KDIGO criteria. Of the 216 patients included, 21 (10%) presented AKI. AKI was significantly associated with age, Mehran score, GRACE score, and NT-proBNP. AKI patients had significantly lower retinal vascular density (RVD)) and had more frequent low RVD (81% vs 45%, P = 0.002). Adding low RVD to the Mehran score and the NT-proBNP, or to the GRACE score and the NT-proBNP, significantly improved their predictive values, suggesting that systemic microvascular involvement remains incompletely addressed by either standard risk scores or factors known to be associated with ICA-induced AKI.

## Introduction

At a time when revascularization by angioplasty has taken a predominant place in the management of acute coronary syndrome (ACS), resulting in a pronounced improvement in patient symptoms and prognosis^1^, acute kidney injury (AKI) related to the injection of iodinated contrast agents (ICA) remains a frequent complication. The incidence of contrast-induced AKI ranges from 10 to 20% of patients who undergo procedures for ACS^2^. AKI is directly correlated with short-term patient prognosis, with a fivefold increase of intrahospital mortality, as well an increased risk of end-stage renal failure in the long term^3–6^. Several studies have made it possible to specify the main determinants of contrast-induced AKI, some of which establish risk scores, such as the Mehran score^7^. Some new risk markers for AKI have recently been identified in ST elevation myocardial infarction (STEMI) patients, such as the severity of coronary artery disease, characterized by a SYNTAX score >10^8^, a GRACE score >160^9^ and a high level of NT-proBNP at admission^10^. The observed actions of ICAs reported so far associate hypoxia-inducing renal medullary vasoconstriction with acute tubular necrosis secondary to endothelin and adenosine release, and direct tubular toxicity^11–13^. Based on the microvascular hypothesis, derived from experimental models of hypertension and diabetes^14–16^, studies analyzing retinal vascularization in patients with chronic kidney disease have demonstrated an independent association between microvascular retinopathy and chronic kidney disease (CKD)^17–21^. While the link between retinal vascular damage and CKD is well documented, no data are available on the relationship between AKI and retinal microvascular dysfunction. However, given the multiple evidence of combined retinal and systemic damage (including myocardial^22^ and cerebral damage^23–25^), and given the microcirculatory involvement in AKI, retinal abnormalities may be associated with the occurrence of AKI after coronary angiography in patients at high cardiovascular risk.

Optical coherence tomography angiography (OCT-A) is a new noninvasive technique based on blood flow imaging. It allows a reproducible study of retinal vascularization through the analysis of erythrocyte movement without fluorescent dye injection^26^. The contrast between static retinal cells and erythrocyte movement provides a model of retinal vascularization^27^. Furthermore, this technique’s software provides automatic quantitative evaluation of retinal vascular parameters such as vascular density. The EYE-MI study, recently conducted in our center, identified an independent association between low retinal vascular density (RVD) with impaired left ventricular ejection fraction (LVEF) and high cardiovascular risk in patients with ACS using OCT-A to analyze retinal vascular density^28^. Based on these observations, we studied the potential value of measuring retinal vascular density in OCT-A as a marker of microvascular dysfunction related to the occurrence of AKI after coronary angiography for ACS.

## Materials and Methods

### Study design and population

This study was based on the prospective follow-up of a cohort of patients hospitalized for ACS in our Cardiology Intensive Care Unit. All patients treated for ACS (unstable angina, ACS without ST-segment elevation or with ST-segment elevation) between 01 September 2016 and 05 March 2017 and who underwent coronary angiography while they were hospitalized were eligible for an OCT-A within the first 48 h after admission. The exclusion criteria were a history of an eye disease (diabetic and vascular retinopathy, age-related macular degeneration, vitreoretinal abnormality), patients on dialysis, patients not affiliated to national health insurance, patients under legal protection, minors, or those who refused to participate.

The study complied with the tenets of the Declaration of Helsinki, the locally appointed ethics committee (Comité de Protection des Personnes (CPP) Est I, Dijon, France) approved the research protocol and written informed consent was obtained from the patient or his/her legal representative. During the OCT-A exams, a cardiologist monitored the patients’ heart rhythm and hemodynamic status.

### Data collection

Patients’ medical records were collected from the obseRvatoire des Infarctus de Côte d’Or (RICO). This regional survey, set up in 2001, collects clinical and biological data from patients hospitalized for ACS, with written consent, in all Cardiology Intensive Care departments in the Côte d’Or region of France. From these data, we selected information pertaining to cardiovascular risk factors, cardiovascular history, history of chronic renal failure, treatments including beta-blockers, angiotensin-converting enzyme (ACE) inhibitors and angiotensin 2 receptor blockers (ARBs) grouped into renin-angiotensin-aldosterone system inhibitors, diuretic treatments and vasopressors, systolic and diastolic blood pressure (BP), and Killip stage. Biological data were also collected: NT-proBNP (N terminal pro-brain natriuretic peptide), troponin peak, creatinine and creatinine clearance (according to CKD-EPI and modified MDRD), and anemia (according to the WHO definition). Anemia was defined according Mehran score definition (Baseline hematocrit value <39% for men and <36% for women).Angiographic data such as the type of angioplasty, the decision to perform coronary artery bypass surgery, the placement of an intra-aortic balloon pump (IABP) and the volume of ICA injected were retained for analysis. The left ventricular ejection fraction (LVEF) was measured with Simpson’s biplane method of discs within the 24 h following admission^29^. The SYNTAX score, an anatomical-based risk score for coronary lesions (length, bifurcation, diffuse disease, calcifications, thrombus, total occlusion) was determined for all patients who received a coronary angiography and for whom the initial SYNTAX score was evaluable^30^. The GRACE admission score assessing the patient’s individual ischemic risk and prognosis with calculation of the probability of in-hospital and 6-month mortality was also calculated^31^.

### Measurement of retinal vascular density and central avascular zone with OCT-A

The study was conducted on 3 × 3-mm angiograms with CIRRUS™ HD-OCT Model 5000 (Carl Zeiss Meditec, Lena, Germany); its technical aspects have been recently described^32^. OCT-A is a rapid noninvasive examination (less than 30 s), and feasible in 82–86% of patients. It is performed by nurses and the acquisition time is about 2 min for each patient. Retinal vascular density and the foveal avascular zone were measured using software for automatic quantitative analysis (Angioplex software v10.0). OCT-A showed substantial reproducibility (intraobserver ICC = 0.86, 95% CI, 0.71–0.94; interobserver ICC = 0.89, 95% CI, 0.81–0.94). Retinal vascular density is the average density of the superficial capillary plexus in the four sectors (superior, inferior, temporal and nasal) initially analyzed separately (Supplementary Fig. 1). Vessel density (length, mm^−1^) represents the total length of perfused vasculature per unit area in a region of measurement.

Similarly, the foveal avascular zone (FAZ, mm²) corresponds to the retinal zone of high cone density and low capillary vessel count, delineated by the perifoveal anastomotic arcade. Given the good correlation between the vascular characteristics of the two eyes demonstrated in several studies on retinal OCT-A^33^, only one eye was retained for analysis: the right eye for the first patient and then the left eye for the second patient for images of equivalent quality and so on for subsequent patients. If the patient was single-eyed, the image was taken from the functional eye. If one of the two images was uninterpretable, the other eye was used. Only images collected with a signal strength >7/10 were retained. Given the absence of vascular density standards or cutoffs on the retinal OCT-A, we determined a threshold value after analysis of the ROC curves for the prediction of AKI, allowing us to define two groups: the first group had a low vascular density while the second had a high vascular density (Supplementary Fig. 2).

### Acute kidney injury

Acute renal failure after injection of iodinated contrast agent was defined according to the KDIGO criteria, with an increase in serum creatinine (ΔCr) of at least 26.5 µmol/L at 48 h of injection or >50% compared to the initial dosage within 7 days after injection of ICA^34^. The occurrence of AKI defined the “AKI” group, while its absence determined the “non-AKI” group.

We also calculated the Mehran score^7^, which includes eight weighted variables (hypotension, intra-aortic balloon pump, congestive heart failure, chronic kidney disease, diabetes, age >75 years, anemia, and volume of contrast) and has been proved to have good predictive performance on the onset of AKI linked to the injection of ICA.

### Statistical analysis

Continuous variables are presented as means (±SD) and medians (interquartile ranges). The dichotomous variables are expressed as numbers (percentages). For continuous variables, normality was verified with a Kolmogorov test. The characteristics of the AKI and non-AKI groups were compared using the Mann-Whitney test for continuous variables and the chi-square or Fisher exact test for categorical variables. All tests were bilateral, and significance was set at P < 0.05.

For multivariate models, factors associated with AKI were assessed using a stepwise backward multivariate regression analysis with an inclusion and exclusion threshold value of 5%. Before the multivariate models were constructed, collinearity between the variables was excluded. The correlation matrix of the multivariate model shows that the correlation between variables (r-coefficient) did not exceed a positive or negative value of 0.25. Since the NT-proBNP values do not follow a normal distribution, a logarithmic transformation was performed. Chi-square improvements and maximum log likelihood stepwise methods, including the Hosmer-Lemeshow goodness-of-fit test, were used to evaluate the regression model. All multivariate models were tested for multicollinearity and were stable: tolerance ranged from 0.69 for the GRACE score to 0.88 for retinal vascular density. The inflation variation factor ranged from 1.14 for retinal vascular density to 1.44 for the Mehran score. To evaluate the discrimination capacity of the different variables on the occurrence of AKI, we constructed ROC curves for the Mehran score, GRACE score, NT-proBNP and perifoveolar vascular density. The best sensitivity and specificity thresholds were determined using the Youden method^35^. The areas under the ROC curve (AUC) were compared using the method described by DeLong^36^. All analyses were performed using the SPSS 22.0 0 software (SPSS, Inc., Chicago, IL, USA).

## Results

### Population

**Figure 1:**
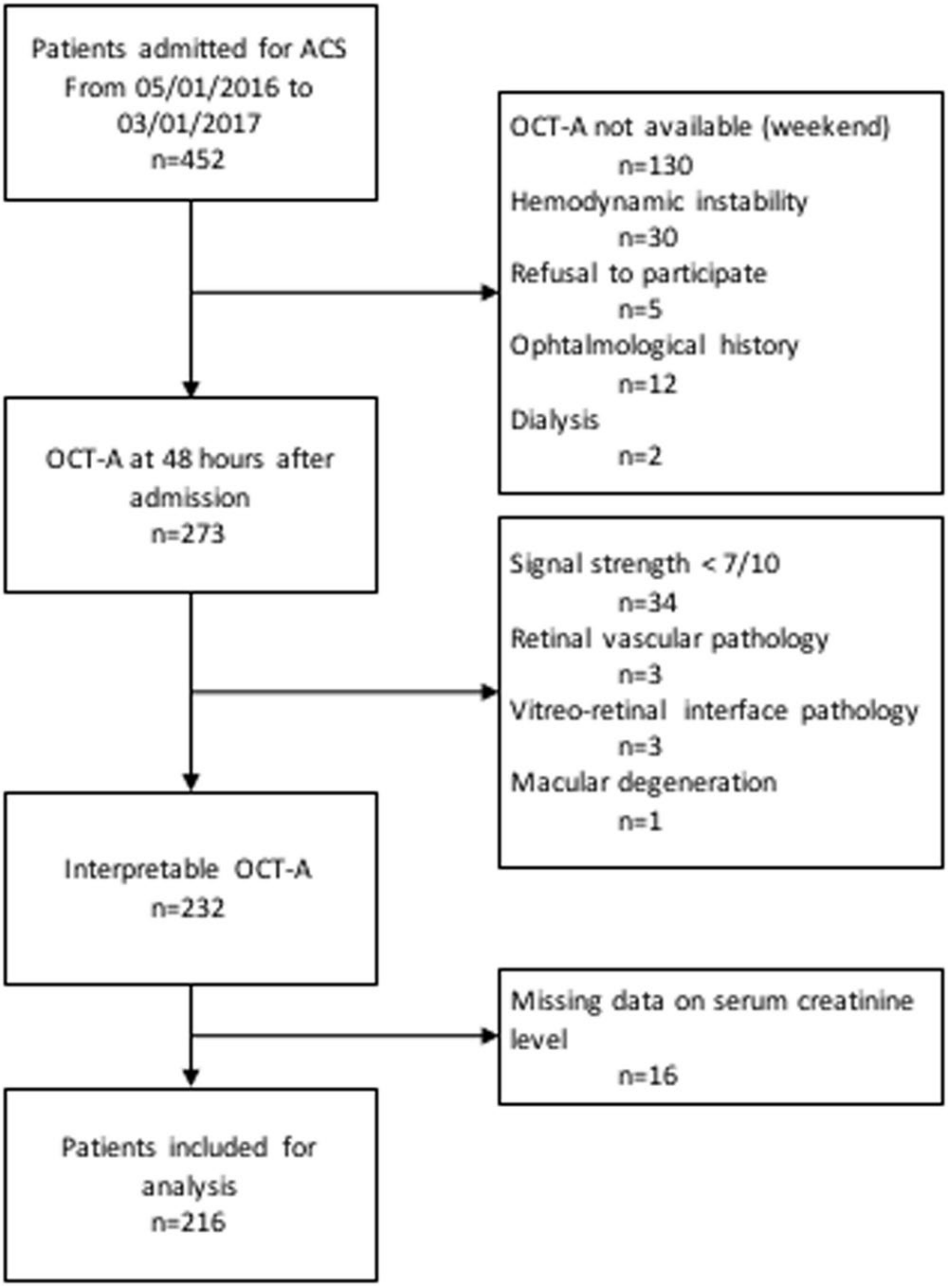
Flow chart.

During the study period, 275 patients hospitalized for ACS in our unit had a coronary angiography and an OCT-A. Finally, 216 patients were included; 21 patients (10%) developed AKI, while the remaining 195 (90%) did not meet the KDIGO criteria (Fig. 1).

### Patient characteristics

The study’s population was predominantly male (79%), with a mean age of 62.6 years. Patients with AKI were significantly older than those who did not develop AKI (69 years versus 62 years, P = 0.009), had a history of atrial fibrillation (19% versus 5.1%, P = 0.014) and chronic kidney disease (19% versus 1%, P < 0.001) (Table 1), and had significantly more symptoms of heart failure on arrival (52.4% versus 15.4%, P < 0.001). The GRACE score was significantly higher in the AKI group (P < 0.001), and biological analyses reported a higher peak troponin and NT-proBNP (P = 0.04 and P < 0.001, respectively) in the AKI group (Table 2 and Supplemental Table).

**Table 1:** Clinical characteristics of the population.

n (%), median (interquartile range), mean (±standard deviation)	AKI (n = 21)	No AKI (n = 195)	P
***Risk factors***			
Age, years	69 (±14)	62 (±12)	0.009
Age > 75 years	9 (43)	28 (14)	0.003
Female gender	4 (19%)	42 (21.5%)	0.791
Hypertension	15 (71.4%)	97 (49.7%)	0.059
Diabetes	5 (23.8%)	46 (23.6%)	0.982
Active smoking	10 (47.6%)	52 (26.7%)	0.093
BMI, kg/m^2^	25.8 (±3.8)	27.2 (±4.8)	0.191
Hypercholesterolemia	9 (42.9%)	78 (40%)	0.8
Familial history of coronary artery disease	5 (23.8%)	70 (35.9%)	0.269
***Underlying heart disease***			
Previous coronary artery disease	4 (19)	40 (20.5)	0.874
Previous heart failure	3 (14.3%)	27 (13.9%)	0.96
Atrial fibrillation	4 (19%)	10 (5.1%)	0.014
*Associated comorbidities*			
Stroke history	1 (4.8%)	6 (3.1%)	0.679
PAD history	1 (4.8%)	9 (4.6%)	0.976
Carotid atheroma history	1 (4.8%)	9 (4.6%)	0.976
Chronic kidney disease	4 (19%)	2 (1%)	<0.001

AKI: acute kidney injury; No AKI: absence of AKI; BMI: body mass index (kg/m²); PAD: peripheral artery disease.

**Table 2:** Clinical, biological and angiographic data.

n (%), median (interquartile range), mean (±standard deviation)	AKI (n = 21)	No AKI (n = 195)	P
***Clinical and echocardiographic data***			
Acute coronary syndrome			0.544
Unstable angina	1 (4.8%)	25 (12.8%)	
NSTEMI	11 (52.4%)	89 (45.6%)	
STEMI	9 (42.9%)	81 (41.5%)	
Killip stage at admission >1	11 (52.4%)	28 (15.4%)	<0.001
Systolic blood pressure, mmHg	140.8 (±40.4)	145.3 (±28.7)	0.513
Diastolic blood pressure, mmHg	82.6 (±21.8)	84.8 (±18.7)	0.608
LVEF (%)	48 (±14)	54 (±10)	0.043
GRACE Score	169 (±38)	127 (±31)	<0.001
Mehran Score	10.1 (±5.5)	4.8 (±3.8)	<0.001
***Treatment at admission***			
Beta-blockers	6 (28.6%)	51 (26.2%)	0.811
RAAS inhibitors	11 (52.4%)	64 (32.8%)	0.074
Diuretics	3 (14.3%)	28 (14.4%)	0.99
***Treatment during CCU stay***			
Beta-blockers	15 (71.3%)	141 (72.4%)	0.932
RAAS inhibitors	14 (66.7%)	150 (76.9%)	0.296
Diuretics	9 (42.9%)	36 (18.5%)	0.009
Vasopressive drugs	4 (2%)	1 (4.7%)	0.430
***Biological data***			
Anemia	7 (33%)	44 (22%)	0.284
Troponin peak, μg/L	75.1 (±96.7)	41.2 (±70.3)	0.045
NT-proBNP, pg/mL	4108.8 (±7021.1)	1296.6 (±3438.3)	0.084
log NT-proBNP	7.4 (±1.6)	5.7 (±1.7)	<0.001
Creatinine, µmol/L	107.3 (±52.3)	78.5 (±20.4)	0.021
Creatinine on day 2, µmol/L	147.5 (±76.6)	83.2 (±18.7)	0.001
eGFR at admission (CKD-EPI), mL/min/1.73 m².	67.8 (±27.4)	87.4 (18.6)	<0.001
eGFR at admission (modified MDRD), mL/min/1.73 m²	75.1 (±33.4)	94.1 (±25.3)	0.018
***Angiographic data***			
Angioplasty	16 (76.2%)	153 (78.5%)	0.811
Aortocoronary bypass surgery	3 (14.3%)	13 (6.7%)	0.205
IABP	1 (4.8%)	0 (0.0%)	0.002
Injected contrast volume (mL)	162 (±75)	146 (±63)	0.249
Initial SYNTAX score	14.2 (±9.1)	11.9 (±9.6)	0.293

AKI: acute kidney injury; CCU: coronary care unit; eGFR: estimated glomerular filtration rate; IABP: intra-aortic balloon pump, LVEF: left ventricular ejection fraction; NSTEMI: acute myocardial infarction without ST segment elevation; NT-proBNP: N terminal pro-brain natriuretic peptide; RAAS: renin-angiotensin-aldosterone system; STEMI: acute myocardial infarction with ST segment elevation.

Regarding coronary angiography data, patients with AKI were more likely to have received IABP (P = 0.002) (Table 2).

Therapeutically, there was no significant difference between the two groups for renin-angiotensin-aldosterone system inhibitors, but patients with AKI had significantly more diuretics at day 2 (P = 0.009).

### Retinal vascular density and AKI characteristics

**Figure 2:**
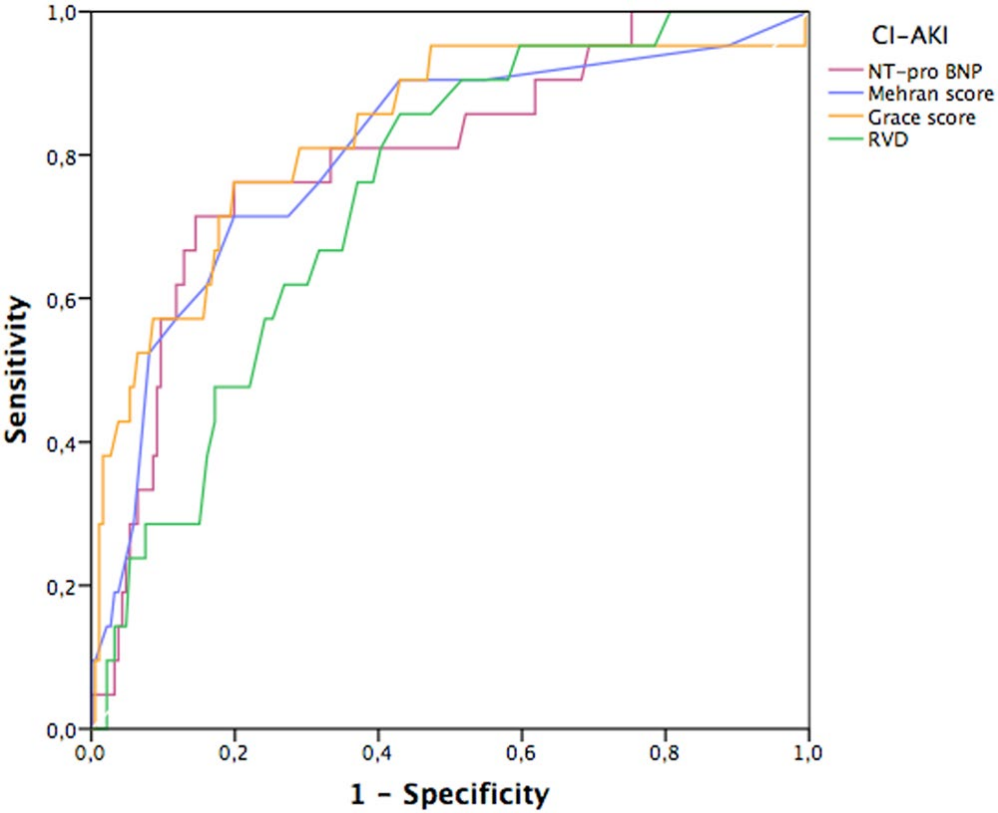
ROC curves comparing sensitivity and specificity of retinal vascular density to the Mehran score, NT-proBNP and GRACE score for the occurrence of contrast-induced AKI.

The mean perifoveolar vascular density (inner vessel) was 17.5 mm^−1^ in the AKI group compared with 19.3 mm^−1^ in the non-AKI group (P = 0.001) (Table 3). Patients in the AKI group were more likely to have low retinal vascular density (81% versus 45.1%, P = 0.002). The AKI group had a significantly higher Mehran score (10.1 versus 4.8, P < 0.001). Analysis of the ROC curves (Fig. 2) showed no difference in performance for the prediction of AKI for retinal vascular density (area under the ROC curve (AUC) 0.745; 95% CI 0.649–0.841; P < 0.001), Mehran score (AUC 0.801; 95% CI 0.696–0.912; P < 0.001), NT-proBNP (AUC 0.794; 95% CI 0.688–0.899; P < 0.001), and the GRACE score (AUC 0.828; 95% CI 0.724–0.932; P < 0.001). The best threshold for retinal vascular density associated with the occurrence of AKI was <19.7 mm^−1^ with a sensitivity (Se) of 81% and a specificity (Sp) of 60%. Analysis of the ROC curves determined the best threshold values of 5 for the Mehran score (Se 76%, Sp 69%), 142 for the GRACE score (Se 81%, Sp 71%) and 512 pg/mL for the NT-proBNP (Se 81%, Sp 66%).

**Table 3:** OCT-A data.

n (%), median (interquartile range), mean (±standard deviation)	AKI (n = 21)	No AKI (n = 195)	P
FAZ (mm²)	0.29 (±0.1)	0.28 (±0.13)	0.737
Inner vessel density, mm^−1^	17.5 (±2.1)	19.3 (±2.3)	0.001
Full vessel density, mm^−1^	16.9 (±2.4)	18.0 (±2.2)	0.065
Inner perfusion density, (unitless, ×100)	0.34 (±0.04)	0.35 (±0.04)	0.168
Full perfusion density, (unitless, ×100)	0.31 (±0.04)	0.33 (±0.04)	0.239

AKI: acute kidney injury; FAZ: foveal avascular zone.

**Figure 3:**
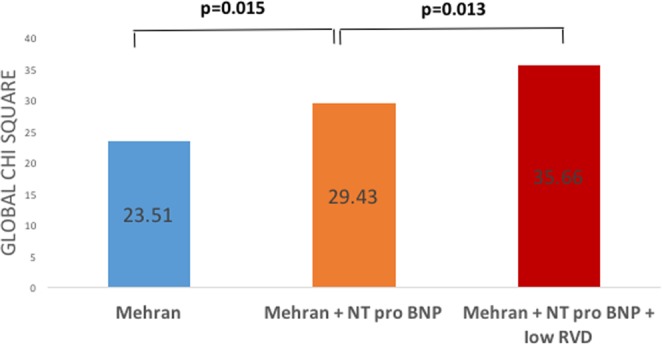
Incremental value of low retinal vessel density (<19.7 mm^−1^) to predict AKI after ACS. Bar graphs illustrating the change in global chi-square value by the addition of low RVF and NT-proBNP level to a logistic regression model including the Mehran score. The addition of NT-proBNP level and low RVD significantly improved the overall chi-square, thereby demonstrating the incremental value of these parameters to predict AKI after ACS.

**Figure 4:**
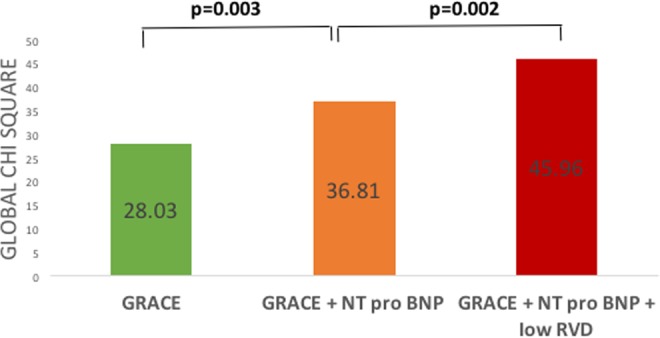
Incremental value of low retinal vessel density (<19.7 mm^−1^) to predict AKI after ACS. Bar graphs illustrating the change in global chi-square value by the addition of low RVF and NT-proBNP level to a logistic regression model including the GRACE score. The addition of NT-proBNP level and low RVD significantly improved the overall chi-square, thereby demonstrating the incremental value of these parameters to predict AKI after ACS.

The objective here was to determine the added value of low vascular density in understanding and preventing contrast-induced AKI. Therefore, the multivariate analysis was carried out on the basis of the two scores classically described as associated with AKI following the injection of a contrast agent (Mehran and GRACE), comparing the improvement in the performance of these scores by the consecutive addition of two variables strongly associated with univariate AKI analysis but that do not figure in the scores and are not collinear: low vascular density defined from the threshold value on the ROC curve (<19.7 mm^−1^) and NT-proBNP (continuous level rather than a cut-off because the predictive value is higher). We therefore established two multivariate models including, first, the Mehran score, NT-proBNP and low vascular density (model 1), and second, the GRACE score, NT-proBNP and low vascular density (model 2). In addition to the statistically significant incremental value added by NT-proBNP to these two scores, the addition of the low vascular density evaluated by OCT-A further improved the performance of the AKI model for model 1 (chi-square = 35.66; P = 0.013) as well as for model 2 (chi-square = 45.96; P = 0.002) (Figs 3 and 4).

## Discussion

To our knowledge, this study is the first to show an association between retinal vascular density and the occurrence of acute kidney injury following the injection of contrast agents. The main results of this study are as follows:

1. Low retinal vascular density (LRVD) is a biomarker for onset of acute kidney injury with iodinated contrast media.
2. LRVD defined as less than 19.7 mm^−1^ is an independent predictor of contrast-induced AKI, and retinal vascular density has a predictive performance equivalent to the Mehran score.
3. The sequential addition of NT-proBNP and low vascular density to the GRACE or Mehran scores significantly improves the ability of these scores to predict AKI.

### Retina and AKI

This prospective observational study shows for the first time that low retinal vascular density assessed by OCT-A is associated with acute kidney injury.

Since the mechanisms of contrast-induced nephropathy are mainly related to microvascular and endothelial dysfunction, the analysis of the potential relationship with OCT-A measurement of retinal vascular density is therefore relevant. Indeed, in a study of more than 3000 patients, Lim *et al*.^21^ demonstrated an association, independent of classic cardiovascular risk factors, between changes in glomerular filtration rate (GFR) and retinal abnormalities (decrease in arterial diameter and alteration of fractal dimension) (P = 0.008). In a case–control study conducted by Sng *et al*.^37^, the fractal dimension was associated with chronic renal failure after adjustment for cardiovascular risk factors (OR 2.47; 95% CI 1.45–4.21). On the other hand, from a cohort of 3199 patients followed for 15 years, the “Beaver Dam CKD” study demonstrated the absence of a causal link between worsening of GFR and retinal vascular abnormalities after adjustment (P = 0.3)^38^. The authors highlighted a parallel injury through shared pathophysiology between retinal and renal disease. These observations were more recently confirmed by Yip *et al*., who analyzed the occurrence of terminal CKD in 5763 patients with an average follow-up of 4.3 years^39^. Thus, retinal OCT-A, which is a noninvasive, rapid, reproducible examination that can be done by paramedical personnel, and whose analysis is automated and instantaneous, appears to be a relevant tool for the evaluation of systemic microvascular involvement.

### AKI and risk scores

The incidence of AKI was 10% in our population, which is comparable to the literature^40^. We used the Mehran score, validated in the ACS population, for its comprehensive multiparametric approach (clinical, biological, angiography procedure parameters). Several studies underlined similar Mehran scores regardless of the AKI definition. In the analysis of the ROC curves, Liu *et al*. reported an AUC at 0.776 (P = 0.219) for an AKI defined by an ΔCr ≥ 0.3 mg/dL (i.e., 26.5 µmol/L), and an AUC at 0.840 (P = 0.247) for a ΔCr ≥ 0.5 mg/dL^9^. Although some of the score parameters in the present study were not independently associated with AKI (notably the volume of injected ICA), the results are consistent with other studies published on the topic.

In our analysis, the GRACE score had a performance close to the Mehran score. Another team previously demonstrated that a GRACE score ≥160 allows AKI to be predicted independently in multivariate analysis (OR 3.84, 95% CI 1.61–9.17, P = 0.002), with a predictive ability similar to the Mehran score (AUC 0.723)^9^. In our series, the threshold value was 142 (Se 81%, Sp 71%), which is in agreement with the threshold of 140 traditionally used to define the population at a high risk of cardiovascular events^31^. These results are explained by the fact that multiple hemodynamic and renal parameters, directly involved in the occurrence of AKI, were taken into account in this model. The ability of the Mehran and GRACE scores to similarly predict the occurrence of AKI as well as major cardiovascular events^41^ indicates that ICA-induced nephropathy may be a marker of events in high cardiovascular risk patients.

### Contributions of NT-proBNP and low retinal vascular density

Previous work carried out in our team by Goussot *et al*. showed that in a STEMI population, with 10.4% evolving toward AKI, NT-proBNP predicted the occurrence of contrast-induced nephropathy significantly in multivariate analysis^10^. Our current study is in agreement with Goussot *et al*.’s work, since NT-proBNP is strongly associated with ICA-induced AKI. In our analysis, the threshold value of 512 pg/mL (Se 81%, Sp 66%) was very close to the data in the literature^42,43^. This biomarker is usually increased in cases of myocardial injury^44^. Hemodynamic renal impairment in the context of ACS is also a factor involved in the increase of this peptide, partly by decreasing its clearance^45,46^. Finally, other factors associated with the increase in NT-proBNP (such as older age, diabetes and CKD) are also predictive factors for contrast-induced AKI. However, NT-proBNP retains an incremental value in the prediction of AKI when coupled with the Mehran and GRACE scores, which in turn directly take into account the patient’s age, kidney function, presence of diabetes, and hemodynamic status.

Low retinal vascular density was strongly associated with AKI in bivariate analysis, and in addition, provided an incremental value for the prediction of AKI when added to the Mehran score (or the GRACE score) and NT-proBNP. It is also known that no-reflow, involving endothelial dysfunction^47^, is associated with an increased risk of acute renal failure^48,49^. All these data suggest a microvascular alteration common to all these organs, leading to their respective dysfunctions. Microvascular abnormalities and endothelial dysfunction (altering renal, retinal and possibly cardiac vascular self-regulation) appear to be imperfectly addressed by standard scores and biomarkers (GRACE, Mehran, NT-proBNP), which may explain the increased value of low retinal vascular density in predicting AKI risk.

These results support the hypothesis that microvascular dysfunction, characterized by low retinal vascular density, could play a role in the occurrence of contrast-induced AKI after coronary angiography in the context of ACS.

## Limitations

There are some limitations to the study. The first is the relatively small number of patients included compared to other studies on AKI with postcoronary angiography ICAs. On the other hand, only patients who could benefit from the ophthalmological examination were included, automatically excluding the most severe patients who could not be moved to the examination room. Third, the “low vascular density” threshold value found is close to the population’s median vascular density, suggesting that this measurement alone cannot be used to stratify the risk of AKI, but instead should be considered in combination with other variables or integrated into a score as suggested here. Quantitative data concerning vascular density should be taken with caution as they were not validated with a gold standard measurement of vessel density in histology^50^.

## Conclusions

AKI was strongly and independently associated with low retinal vascular density. OCT-A evaluation of retinal vascular density offers a noninvasive, reliable and rapid result supporting the hypothesis of systemic microvascular involvement directly contributing to the occurrence of contrast-induced AKI. The presence of a low retinal vascular density even seems to predict AKI with diagnostic performance close to standard prediction scores, and the incremental value of this new biomarker suggests that microcirculatory damage has not been taken into account in determining AKI risk until now. While prevention methods are still limited, identifying the mechanisms and establishing strong predictive factors are key steps towards the prevention of contrast-induced nephropathy after myocardial infarction.

## Supplementary information

Supplementary figures and table

## Data Availability

All relevant data are within the paper. Additional data available upon request.
