# Supplementary material for: Retinal Vascular Density as A Novel Biomarker of Acute Renal Injury after Acute Coronary Syndrome
Source: Sci Rep. 2019 May 30;9:8060. doi: 10.1038/s41598-019-44647-9 (PMC6543041; doi:10.1038/s41598-019-44647-9)
Supplement: Supplementary file 1 — Supplementary figures and table [file 41598_2019_44647_MOESM1_ESM.docx]

**RETINAL VASCULAR DENSITY AS A NOVEL BIOMARKER OF ACUTE RENAL INJURY AFTER ACUTE CORONARY SYNDROME**

Guillaume Alan*, MD ^a^, Charles Guenancia*, MD, PhD ^a,b^, Louis Arnould, MD, MSc ^c,d,e^, Arthur Azemar, MD ^a^, Stéphane Pitois, MD ^a^, Maud Maza ^b^, Florence Bichat, PhD ^b^, Marianne Zeller, PhD ^b^, Pierre-Henri Gabrielle, MD, MSc ^c,e^, Alain Marie Bron, MD^c,e^, Catherine Creuzot-Garcher, MD, PhD ^c,e^, Yves Cottin, MD, PhD ^a,b^

^a^ Cardiology Department, University Hospital, Dijon, France

^b^ PEC 2, Univ. Bourgogne Franche-Comté, Dijon, France

^c^ Ophthalmology Department, University Hospital, Dijon, France

^d^ INSERM, CIC1432, clinical epidemiology unit, Dijon, France; Dijon University Hospital, Clinical investigation Center, Clinical epidemiology/clinical trials unit, Dijon, France

^e^ Eye and Nutrition Research group, CSGA, UMR 1324 INRA, Dijon, France

*These authors contributed equally to this work.

**Corresponding author**:

Charles Guenancia, Cardiology Department, University Hospital, 14 rue Paul Gaffarel, 21079 Dijon CEDEX, France.

Tel/Fax: +33380293536/+33380293879

Email: [charles.guenancia@gmail.com](mailto:charles.guenancia@gmail.com)

**Funding:** This work was supported by the Dijon University Hospital, the Association de Cardiologie de Bourgogne, and by grants from the Agence Régionale de Santé (ARS) de Bourgogne, French Ministry of Research, Institut National de la Santé et de la Recherche Médicale (INSERM), Fédération Française de Cardiologie, Société Française de Cardiologie and the Regional Council of Burgundy.

####
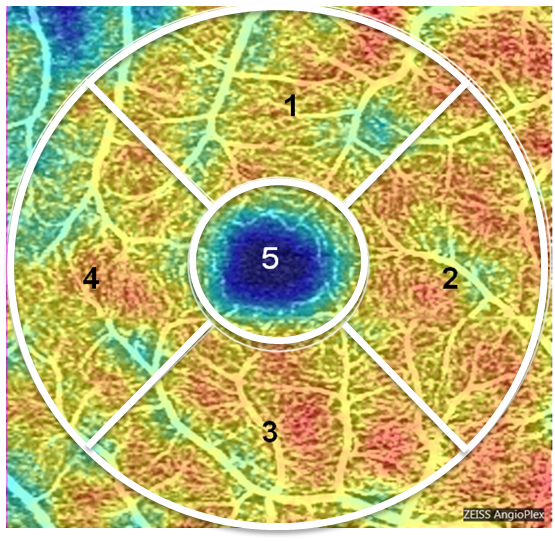


#### **Supplementary Figure 1.** Retinal OCT-A segmentation: four sectors (1–4) and foveal avascular zone (FAZ) (5).


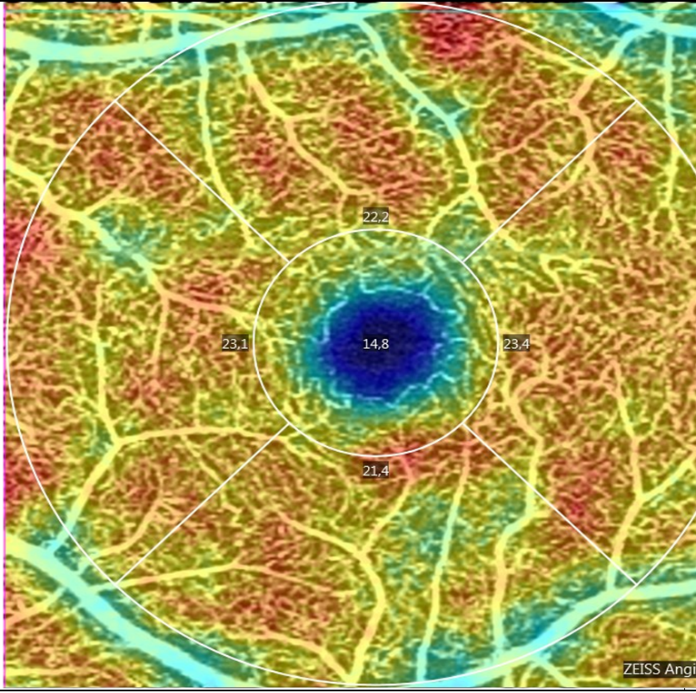

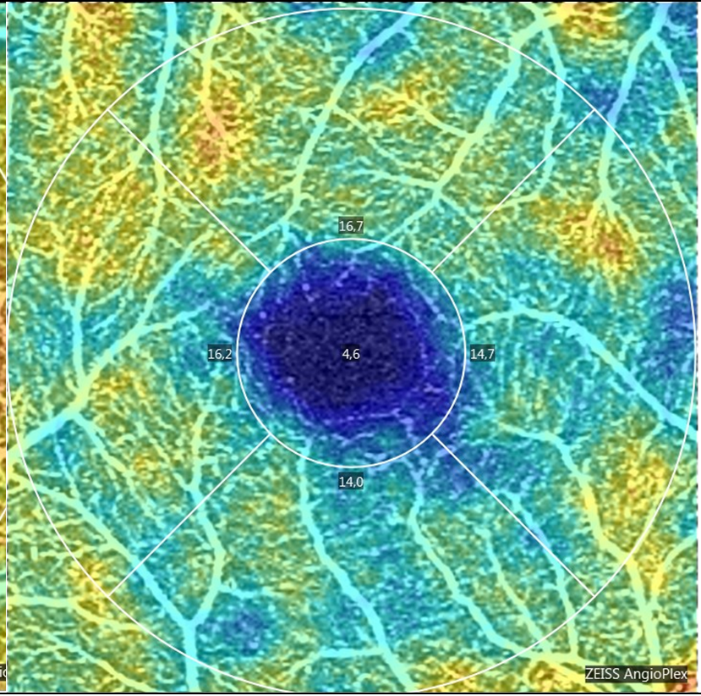


**A**

**B**

**Supplementary Figure 2.** Example of vascular density. A: patient from low vascular density group. B: patient from high vascular density group.

**Supplemental Table: Bivariate logistic regression analysis for predictors of acute kidney injury in acute myocardial infarction patients (n=223).**

| **Variable** | **HR** | **95%CI** | **p** |
| --- | --- | --- | --- |
| Age >75 years | 4.47 | 1.73-11.6 | 0.002 |
| Diabetes | 1.01 | 0.35-2.91 | 0.982 |
| Congestive acute heart failure | 7.63 | 2.95-19.74 | <0.001 |
| Anemia | 1.72 | 0.65-4.52 | 0.274 |
| Chronic kidney disease | 22.71 | 3.87-133.08 | 0.001 |
| NT pro BNP>512 pg/mL | 8.12 | 2.63-25.1 | <0.001 |
| Creatinine at admission (µmol/L) | 1.03 | 1.01-1.04 | <0.001 |
| Low RVD (<19.7 mm^-1^) | 5.17 | 1.68-15.92 | 0.004 |
| Injected contrast volume (mL) | 1.004 | 0.99-1.01 | 0.249 |

CI: confidence interval; HR: Hazard Rate; RVD: retinal vascular density
